# Association between Anxiety, Quality of Life and Academic Performance of the Final-Year-Students in Latvia

**DOI:** 10.3390/ijerph18115784

**Published:** 2021-05-27

**Authors:** Inta Zile, Ieva Bite, Indra Krumina, Valdis Folkmanis, Lilian Tzivian

**Affiliations:** 1 Faculty of Medicine, University of Latvia, LV-1004 Riga, Latvia; inta.zile@lu.lv (I.Z.); valdis.folkmanis@lu.lv (V.F.); 2 Faculty of Psychology, University of Latvia, LV-1586 Riga, Latvia; ieva.bite@lu.lv; 3 Kauguri Health Center, LV-2016 Kauguri, Latvia; indra.krumina@gmail.com; 4 Holon Institute of Technology, Holon 5810201, Israel

**Keywords:** level of anxiety, students’ quality of life, high-rated schools, gender differences

## Abstract

The main objective of this study was to investigate the association between final-year students’ anxiety level and quality of life (QOL) with their academic achievements. A longitudinal study was performed in regular schools and in high-rated gymnasiums at the beginning and at the end of the school year. Multiple linear regression models were built for the association between level of anxiety/QOL with academic achievements. Type of school and gender—but not the level of anxiety—were the main predictors of academic achievements of 287 adolescents (e.g., for mathematics, the effect estimates were: β = −1.71 [95% confidence interval −2.21; −1.21]; β = −0.50 [−0.95; −0.06], β = 0.09 [−0.02; 0.20] for the type of school, gender, and changes in level of anxiety, respectively). To conclude, particular efforts should be made to reduce the level of anxiety in girls, especially those that study in high-rated schools.

## 1. Introduction

Anxiety disorders are one of the most common types of psychiatric disorders in adolescents. In the last World Health Organization (WHO) report on adolescents’ mental health, anxiety was ranked in the five most common causes of disability-adjusted life years (DALYs) in 10–19 years old adolescents, leading to 430 DALYs in those aged 10–14 and to 532 DALYs in those aged 15–19 [1]. Relationships between anxiety and academic performance of students has been investigated for a long time, but information on this relationship is still controversial. Most studies have reported an inverse association between anxiety and academic performance. Thus, in the meta-analysis published in 1991 by Seipp, which analyzed 26 publications, an association between higher level of anxiety and lower academic performance was found [2], especially for cognitively determined and specific anxiety measures. However, in other studies higher anxiety was related to a better academic performance [3], especially for highly talented students. 

This corresponds to the Yerkes-Dodson law first described in 1908 [4]. According to this law, elevated arousal levels can improve performance up to a certain point, and after that point, an arousal becomes excessive and diminishes performance. It is additionally known that students with good and very good academic grades have a lower level of anxiety than those with insufficient grades [5]. Anxiety also increases in students corresponding to the level of education, ranging from 2.3% of anxious students in elementary school to 15.9% in high schools [5]. 

One of the factors that has been found to be related to the level of anxiety is the health-related quality of life (QOL). People with anxiety disorders were found to have a reduced QOL [6]. Association between anxiety and QOL was found for different populations both in cross-sectional [7,8,9,10,11] and in longitudinal [12] studies. However, the studies on QOL in healthy adolescents are scarce. QOL of adolescents is a subjective measure that assesses physical and emotional functioning, social functioning, and school functioning [13]. QOL of students can predict children with potential problematic behavior and help to identify those who need help from mental health agencies. Psychological, emotional, and social domains of QOL were shown to be related to a level of anxiety of students [14]. In adolescents with high anxiety level, poor QOL was constated in all QOL domains [15,16]. 

In Latvia, the problem of adolescents’ mental health is especially acute. For example, in the large study by Ravens-Sieberer et al. (2009), Latvia had the lowest life satisfaction among 41 investigated countries. In connection to the poor life satisfaction, Latvia also showed a high percentage of adolescents with multiple health complaints [17]. 

This data did not change in the recent research report on well-being of young people in Baltic states [18]. This report shows that 30% of Latvian adolescents have low self-perceived health; 7% of Latvian adolescents are depressed, and this corresponds to a high level of adolescents’ suicides (11 per 100,000 for those aged 11–15). It is noteworthy that in a similar report two years earlier, in 2017 [19], 25% of 15-year-old adolescents rated their health as poor, and among girls this was 38%. All these data make investigations of 15-year-old adolescents’ mental health in Latvia extremely important. However, we did not assume particular differences in psychological factors between adolescents of Latvia and those from other European countries, making findings of this study important for all European educational authorities.

The main aim of our study was to investigate the association between the level of anxiety, QOL, and academic achievement in final-year students at regular secondary schools and high-rated gymnasiums, which give secondary education on higher level and require higher academic achievements in all subjects before admission. Assuming, on one hand, possible better academic performance of students at high-rated gymnasiums in comparison with regular schools, and, on the other hand, possibly more prominent problems in mental health and quality of life in students learning at high-rated gymnasiums, we posed several questions that can shed light on the necessity of existence of these high-rated gymnasiums. Specifically, our questions were as follows:

1. Do the students of high-rated gymnasiums achieve better marks during the final state examinations?
2. Are students of high-rated gymnasiums more anxious than the students at the regular schools, and does this level of anxiety change during the final school year?
3. Is there any difference between students of high-rated gymnasiums and students at regular schools in their QOL?
4. Can the gender differences explain differences in marks and in the level of anxiety before exams? Are there gender differences in QOL of final-year students?
5. Is the level of anxiety an independent predictor of students’ marks at the beginning of the school year and at the state exams?

We hypothesized that students of high-rated gymnasiums will display higher academic performance than those from the regular schools. At the same time, we further hypothesized that the level of anxiety of these students will be higher than that of the students from regular schools. We assumed that the level of anxiety will increase before a state exam, thus affecting students’ academic performance. We assumed QOL of students at regular schools to be different from students of high-rated gymnasiums. Last, we expected girls to have a higher level of anxiety than boys, which would subsequently affect their grades. We supposed level of anxiety, gender, and the type of the school independently to predict students’ academic performance. 

## 2. Materials and Methods

### 2.1. Measurements

#### 2.1.1. Study Design and Population

Participation in the study was proposed to eight schools in Riga, Latvia; of them four were high-rated gymnasiums, and another four were regular secondary schools according to accepted categories in Latvia. Due to a suicide attempt that took place in one of the gymnasium schools right before the beginning of the survey, this school was excluded from the study, thus reducing the number of gymnasiums included in the study to three. Parents and students received an explanation of the aims of the study and its importance and were asked to sign an informed consent document. The study was reviewed by the Research Ethics Committee of the University of Latvia. Baseline investigation started at the beginning of November 2018, and a follow-up investigation was performed at the end of April 2019 for all students whose parents had signed the informed consent document. Dates of investigation were considered according to the possible level of anxiety that a student might have at the beginning of the school year after the first recognition of the school rules (a low level according to our assumptions) and before the state exam (higher level). Demographic and school-related variables were assessed at baseline. We assessed students’ age, gender, and school type. We performed two-point cross-sectional analyses of obtained data.

#### 2.1.2. Academic Achievement

At the end of the school year, ninth grade students take a final exam in three subjects: Latvian language (native language), foreign language (English, German, Russian or French), and mathematics. Students with higher academic achievement during the state exams will have the possibility to study in better secondary schools. In the case of poor academic achievement, the level of school can be reduced from a gymnasium to a regular secondary school or even to low-rated professional schools. In some cases, students with extremely low achievement will not be given the possibility of continuing their education and will start their unqualified work. To prepare the students for these exams and with the attempt to understand weakness in students’ knowledge, schools perform mid-term tests at the end of the first study semester. Obviously, these mid-term tests are less stressful for students and check approximately half of the knowledge that will be checked during a final state exam. According to Latvian rules, academic achievement can range between 1 (worst performance) and 10 (best performance), and the minimal passing score for the exam is 4. Children can avoid participation in the final state exams due to medical or other reasons, and in this case the mean academic achievement of two semesters is counted as the final score. 

We checked students’ academic performance in all three subjects (Latvian language, foreign language, and mathematics) at two points: at the end of the first semester (school exams), and at the end of the study year at the final centralized state exams. 

#### 2.1.3. Level of Anxiety and QOL

Level of anxiety of students was assessed using the Achenbach System of Empirically Based Assessment (ASEBA) translated into Latvian. This inventory consists of 112 questions that assess different psychological conditions, such as affective disorders, anxiety, somatic complains, attention deficit problems, oppositional challenge disorders, and behavioral problems. We used the ASEBA DSM-oriented anxiety scale with six questions to assess the level of student anxiety characterized by dependence on adults, overall fear, fear of school, tension, self-reported level of anxiety, and worries [20]. Answers for each question ranged from 0 (do not conform/match) to 2 (totally match), and a sum of all answers displayed the overall anxiety of each student. 

The possible range for anxiety was from 0 to 12, with higher values displaying higher levels of anxiety. All points were summarized and compared with ASEBA norms in Latvia, thus additionally dividing the subjects according to gender norms described in ASEBA. For boys, a level higher than five, and for girls, a level higher than six indicated a high anxiety level, and levels below these values grouped together non-anxious participants and those with low anxiety. 

QOL was assessed using the SF-36 inventory [21] translated into Latvian (possible range of answers from 0 to 100). Mean scores were calculated for two to ten questions of the survey according to questionnaire recommendations to assess QOL-specific domains: physical functioning (ten questions), role limitations due to physical health (four questions), role limitations due to emotional problems (three questions), energy/fatigue (four questions), emotional well-being (five questions), social functioning (two questions), pain (two questions), and general health (five questions). The possible range for each one of the QOL domains was from 0 to 100. 

### 2.2. Data Analysis

#### 2.2.1. Descriptive Statistics and Comparisons between Groups

Descriptive statistics were performed for all study variables. We assessed means and standard deviations for normally distributed variables, and medians, minimal, and maximal values, and inter-quartile range (IQO) for variables with distributions other than normal. For categorical variables, we presented frequencies and percent. 

The reliability of answers on anxiety and QOL questions was investigated using α-Cronbach test. Differences between the first semester and the final state exam in students’ academic achievement were obtained using paired t-test, and differences in students’ level of anxiety and in QOL domains during baseline and the follow-up examination were investigated using the Wilcoxon test. In addition, we compared differences in students’ academic performance, level of anxiety, and QOL domains between regular schools and gymnasiums, and between boys and girls using the t-test and the Mann–Whitney test. We compared the percentage of highly anxious students (level of anxiety higher than five for boys and higher than six for girls) between study time points (baseline and follow-up) using the McNemar test, and between types of schools and genders using the chi-square test.

Furthermore, we investigated the univariate association between level of anxiety and students’ academic achievement, using the Spearman correlation. We checked the correlation for the academic achievement of first semester and the final state exam, using anxiety levels from baseline and follow-up time points, respectively.

#### 2.2.2. Multivariate Regression Analysis

Assuming gender, type of the school, and level of anxiety as possible predictors for students’ grades, we built multiple linear regression models for students’ academic achievement in all three subjects for the first semester and for the final state exam individually. We included the level of anxiety in models even if the univariate correlation was not found between it and the investigated academic achievement, as our hypothesis considered it as a primary exposure of interest. 

We performed a sensitivity analysis including a dichotomized variable of level of anxiety (anxious/non-anxious) in the multiple linear regression models to investigate the stability of observed associations. Effect estimates β and 95% confidence intervals (CI) were presented for these models. All analyses were performed using Statistical Package for Social Sciences (SPSS) version 26 [22]. *p* values ≤ 0.05 were considered statistically significant. 

## 3. Results

### 3.1. Study Population

At the beginning of the study, participation was proposed to 493 parent–student pairs from four high-rated gymnasiums and four regular schools. After the explanation of goals of the study, 305 (61.9%) parents agreed to participate in the study. After excluding one gymnasium as a result of attempt of suicide that took place there before the beginning of the study, the reduced final study sample was 287 adolescents, of which 207 (72.1%) were from gymnasiums and 80 (29.9%) from regular schools. Almost one-half of the participants were girls (N = 141, 49.1%). There were no gender differences between regular secondary schools and gymnasiums (*p* = 0.39). Mean and median age of participants was 15.0 years (standard deviation, SD 0.34; IQR 15; 15), and most participants (72.8%) were of this age. 

### 3.2. Academic Achievement

For different reasons, 15.3% of all children did not participate in the state exam in Latvian language, 14.6% in foreign language, and 15.7% in mathematics. The highest academic achievement of students was observed for foreign language (median for the first semester was 8; and for the final exam was 9, respectively). In other subjects, the range of medians was 6–7 for semester academic achievement and for the final exam. Almost all academic achievement was normally distributed according to skewness and kurtosis tests (both <1 for all subjects) and histograms. The only academic achievement that had slight deviation from normal distribution was that of the state exam in foreign language (skewness −1.15, kurtosis 1.15). Nevertheless, we decided to use parametric methods for all academic achievement to unify the methodology. We observed statistically significant differences between academic achievement in the first semester and on the final exam in all subjects. For Latvian and foreign languages, the final exam was higher than that of the first semester’s, but in mathematics first semester achievement was higher than that of the final exam (Table 1). 

There was statistically significant difference between regular secondary schools and gymnasiums in academic achievement for all three subjects: students of gymnasiums constantly displayed higher results in all subjects (Table 2). 

Girls scored significantly higher than boys for Latvian language and for mathematics but did not differ in foreign language (Table 3). 

Medial significant correlation was observed in academic achievement for the first semester: rs = 0.50–0.78 assuming better performance in all subject matter for the same students. However, for the final state exam this correlation was weaker: rs = 0.28–0.50 (Appendix A Table A1).

### 3.3. Level of Anxiety and QOL

#### 3.3.1. Reliability of Questionnaires

The reliability of the anxiety questionnaire was medium in both baseline and follow-up (α-Cronbach > 0.7). All QOL domains displayed medium to high internal consistency (α-Cronbach from 0.65 to 0.85) (Appendix A Table A2).

#### 3.3.2. Anxiety Level

The mean level of anxiety was 3.98 (SD 2.56) and 3.78 (SD 2.37) at baseline and follow-up, respectively. There were no statistically significant differences between the level of students’ anxiety at baseline and at follow-up (*p* = 0.08) (Table 1). Most of the children were not highly anxious either at baseline or follow-up (69.0% and 68.6%, respectively), without statistically significant difference between time points. From those who were anxious, more students were from gymnasiums and were girls at baseline; however, these differences disappeared at follow-up (Table 4). 

The level of anxiety differed by school at baseline with higher levels for gymnasiums (*p* = 0.05); however, this difference disappeared in the follow-up (*p* = 0.60). Girls had significantly higher level of anxiety than boys (*p* < 0.01).

The mean level of anxiety decreased before state exams in comparison with the baseline level (0.23 ± 2.05). The range of changes in the level of anxiety was from −5 to 6. For 35.5% of students, the level of anxiety decreased; for 57.0%, it did not change; and for the rest of the students, this level increased. As a result, 28 (12.3%) of students went from anxious to non-anxious; 170 (74.6%) did not change their levels; and 30 (13.2%) changed their status from non-anxious to anxious according to gender norms. 

#### 3.3.3. QOL

For QOL, the highest level was 100 for all domains; however, the lowest point differed. For the baseline, it was 0.0 for role limitations due to physical health, role limitations due to emotional problems, and social functioning; 5.0 for energy/fatigue and general health, 8.0 for emotional well-being, and 12.5 for pain. For the follow-up, two QOL domains had different lowest values: emotional well-being (lowest value 12.0) and general health (lowest value 15.0). None of the QOL domains were normally distributed. Statistically significant differences between baseline and follow-up were observed for energy/fatigue and emotional well-being (*p* < 0.01 for both domains) (Table 1). 

We did not observe any statistically significant differences between schools in any domain of QOL of students either at baseline or at follow-up. Girls had significantly lower QOL for all domains, both at baseline and follow-up (*p* < 0.01 for all domains both at baseline and for follow-up) (Table 2 and Table 3). 

#### 3.3.4. Correlations between Anxiety and Academic Achievement

The level of anxiety significantly positively correlated with semester academic achievement in all subject matters in the first semester (*p* < 0.01) assuming better academic performance for more anxious children. However, all correlation coefficients in these cases were weak (rs from 0.24 to 0.31). For the final state exam, this weak positive correlation between the level of anxiety and academic achievement was observed only for Latvian language (rs = 0.18; *p* < 0.01) but not for the foreign language (*p* = 0.17) and not for mathematics (*p* = 0.24) (Appendix A Table A1).

### 3.4. Multivariate Regression Analysis

In two-point cross-sectional analysis using fully adjusted multiple linear regression models, we did not observe any association between the level of anxiety and students’ academic achievement in subjects considered either for the semester or for the final state exam. The factor that consistently predicted student performance was the type of school: students from gymnasiums were more successful in all subjects (Table 5). Boys were less successful than girls in Latvian language at the first semester (effect estimate β = −1.11 [95% confidence interval −1.60; −0.63]); these gender differences slightly decreased in the final state exam (β = −1.05 [−1.38; −0.71]) and when using a difference in the level of anxiety as a predictor (β = −1.01 [−1.34; −0.67]. Association with gender had an even stronger reduction between first semester and final exam for the exam in mathematics with the lowest point being when taking the difference in anxiety as a predictor (Table 5). There was no difference between genders in foreign language. Although prediction possibilities of all models were low, they were higher for the first semester academic achievement (adjusted R2 range 0.08–0.27; than for the final state exam (adjusted R2 range 0.07–0.21) (Table 5). 

Sensitivity analyses using a dichotomized anxiety variable did not change the significance of predictors of students’ academic achievement showing a stability of observed associations (data not shown). For example, for the final state exam in mathematics, effect estimates were β = 0.05 [−0.02; 0.12] for the continuous anxiety variable and β = −0.24 [−0.55; 0.50] for the dichotomous one. For other variables in these regressions effect estimates, significance levels did not change in any regression models.

## 4. Discussion

At the beginning of the study, we hypothesized that students at high-rated gymnasiums will display higher academic performance than those of regular schools. We observed these differences both during the 1st semester and at the state exams. We observed as well that the level of anxiety of students at high-rated gymnasiums was higher than that of students at regular schools, which corresponds to our hypothesis. We observed the increase in the level of anxiety before the state exams in both types of schools, not specifically in the high-rated ones. However, contrary to our hypothesis we did not observe that the level of anxiety affects ninth grade students’ academic performance at any of the time points. We observed statistically significant differences in energy/fatigue and emotional well-being of students at the beginning and at the end of the school year. The type of school (high-rated gymnasiums and regular schools) and gender were the main predictors in students’ academic achievement in all three subjects at both study points as well as for the difference of achievement between points 

Association between type of school and level of anxiety has been shown in previous studies [23,24], and anxiety affected students’ grades more in regular than in high-rated schools. The level of anxiety in these studies was higher in regular schools and was associated with poorer performance. In contrast, the study by Abu Bakar and Ischak (2014) reported that so-called “gifted students” with a higher level of linguistic abilities and complex problems solving were more at risk for psychological problems including anxiety than students from regular schools [25]. 

Higher school demands strongly correlated with higher level of anxiety (r = 0.71) in the study by Wiklund et al. (2012) [26]. Another study conducted by Fehm & Schmidt (2006) reported an extremely high level of anxiety between music school students. One-third of these students reported a clinical level of anxiety; however, positive or negative correlation between anxiety and academic performance in this study was dependent on individual experiences of participants [3]. All these studies are cross-sectional and can suffer from reversed causality; therefore, the results are inconsistent. 

In the present study, we observed a higher level of anxiety in gymnasiums than in regular schools. This can be explained by high perfectionism of students from gymnasiums that makes them anxious at the beginning of the study year, as well as being placed in a new situation where students still did not know their teachers and the rules of the school. However, in our study these differences disappeared during the school year, and the level of anxiety was similar between school types. This might be a result of high achievement of gymnasium students in the first semester, which makes them more self-confident for the rest of the study year. Both these assumptions were supported by the highest values of reported anxieties in our study. For gymnasiums, the highest value decreased from 10.0 at baseline to 8.0 in follow-up. On the other hand, it might be the result of an increasing level of anxiety of students at regular secondary schools before the final state exam, although this suggestion was not supported by our data. 

Previous studies have constantly shown girls to be more anxious than boys [27,28,29], and our study confirms these findings. Girls have greater difficulties regulating their negative emotions, which subsequently impacts the level of anxiety [30]. According to the study by Eum and Rice (2011), nearly 50% of the variance in anxiety can be attributed to gender [27]. Girls also complain more about pressure from school and excessive school demands. For example, 63.6% of girls and 38.5% of boys experienced high pressure from school in the cross-sectional study performed among 16- to 18-year-old adolescents of Northern Sweden [26]. 

Academic competences and regulations were among the factors increasing the level of anxiety from moderate to high for girls, but not for boys [31]. We did not find the level of anxiety significantly associated at a multivariate level with academic performance either for boys or girls. It is possible that girls who are highly goal-orientated and hardworking can overrate their difficulties during the school year thus attenuating gender differences in the level of anxiety at the time of the final state exam.

In this study we observed higher levels of in energy/fatigue and emotional well-being of students at the beginning and at the end of the school year. Similar results were obtained in a study investigating the QOL of adolescents in 41 European and North American countries and Israel that surveyed 200,000 children (50.9% female) [17]. This study aimed to examine cross-cultural differences in school children’s subjective health, symptom load, and quality of life. In this study, 15-year-old children from all countries had lower life satisfaction and lower general and subjective health than children two years younger. The study by Wiklung et al. described similar results: adolescents reported a decreased physical QOL [26]. This can be related to higher demands of secondary schools in comparison to primary schools. In our study we did not observe differences between the two types of secondary schools in students’ QOL. However, in both school types, girls had worse QOL than boys. Similar data was found in the Lithuanian study by Duèinskienë et al., in which gender differences were found to be most prominent in the physical health QOL domain [32]. This can be related to a higher level of perfectionism of girls that was found, for example, in the study by Eum and Rice (2011) [27]. Treatment of adolescents with high level of anxiety is extremely important in Eastern European countries, and especially in Latvia, where the level of suicide for young adults (age 20–24) is 16 per 100,000 in comparison to 11 per 100,000 at age 15–19 [18]. 

### 4.1. Limitations of the Study

This study has several limitations. First, we compared schools in one city of the country. Although Riga is the capital of Latvia, and about a half of the country’s population lives there, and study programs are the same for all cities in the country, there still might be differences between students in different parts of the country. Secondly, not all schools of Riga were included in the study. The inclusion depended on the level of cooperation of school authorities, which certainly can lead to selection bias. Third, we did not measure the proper socio-economic status of our students. It is known that socio-economic status may affect many parameters including academic performance and QOL. As school education in Latvia is free, the type of the school cannot be a strong proxy of socio-economic status of parents of students. However, it can be an academic factor, as some school facilities can cost more for gymnasium students than for students in regular schools (e.g., excursions and some study materials). The relatively small number of students from the regular schools can reduce the power of performed analyses and limit comparison between types of schools. Assuming the possibility of students’ performance as the true determinant of one of the variables entered in the regression model, as well as a possibility of correlation between independent variables (level of anxiety, gender, type of school), a possibility of omitted variable bias is another possible limitation of this study. The lack of possibility to confirm a causality in such observational cross-sectional study, as well as the limited power of sensitivity analysis using the dichotomized anxiety level are additional limitations of this study. Lastly, the frames of the study do not allow us to make final conclusions about the level of anxiety and academic performance of students in the future. There is a possibility that these parameters will change when students begin higher education in universities, and students at regular schools will be not less successful than those completing their education in highly rated gymnasiums.

### 4.2. Strengths of the Study

To our knowledge, this is the first study that included all three possibly inter-related factors of students’ achievement: level of anxiety and students’ academic achievement. We additionally strengthened the position of other authors that the level of anxiety does not decrease the final grade of students. Although girls were more anxious than boys, and students from high-rated gymnasiums were more anxious than those from regular schools, both genders and students from different types of schools have the same opportunity to succeed.

## 5. Conclusions

The type of the school and gender, but not the level of anxiety predicted academic achievement. According to the results of our study, it is important to identify adolescent girls with high levels of anxiety to improve their coping skills. Girls need to plan regular meals and physical activities, and regulate their sleep regime and use of electronic devices. Hobbies are highly recommended. As the next level of compensatory actions for improving the regulation of anxiety, a psychoeducation, cognitive-behavioral therapy (for example, mindfulness, relaxation techniques, anxiety management, social skills training), dialectical–behavioral therapy, and/or supportive group or individual psychotherapy can be proposed. In the most difficult cases a prescription of antidepressants may be considered. Parents of final-year adolescents should be trained to identify signs of students’ anxiety as early as possible. 

## Figures and Tables

**Table 1:** Descriptive statistics of students’ marks, anxiety level, and QOL, by time point.

Student’s Marks	1st Semester, Mean ± SD	Final Exam, Mean ± SD	*p* Value
Latvian language	6.16 ± 1.52	6.81 ± 1.35	<0.01
Foreign language	7.70 ± 1.28	8.33 ± 1.36	<0.01
Mathematics	6.32 ± 1.77	6.52 ± 1.80	0.01
**Anxiety Level and QOL**	**Baseline** **Median [min; max]**	**Follow-Up** **Median [min; max]**	
Level of anxiety	4.0 [0.0; 11.0]	4.0 [0.0; 10.0]	0.08
Highly anxious (N, %)	61 (21.3)	59 (20.6)	
Physical health	95.0 [20.0; 100.0]	95.0 [20.0; 100.0]	0.74
Role limitations due to physical health	75.0 [0.0; 100.0]	75.0 [0.0; 100.0]	0.79
Role limitations due to emotional problems	66.7 [0.0; 100.0]	66.7 [0.0; 100.0]	0.39
Energy/fatigue	60.0 [5.0; 100.0]	55.0 [5.0; 95.0]	<0.01
Emotional well-being	68.0 [8.0; 100.0]	64.0 [12.0; 100.0]	<0.01
Social functioning	87.5 [0.0; 100.0]	75.0 [0.0; 100.0]	0.15
Pain	77.5 [12.5; 100.0]	77.5 [12.5; 100.0]	0.99
General health	70.0 [5.0; 100.0]	65.0 [15.0; 100.0]	0.14

**Table 2:** Differences in grades, anxiety, and QOL, by schools.

	Regular School	Gymnasium	*p* Value	Mean Difference	95% CI
Latvian language, mean ± SD					
1st semester	5.68 ± 1.51	6.46 ± 1.45	<0.01	0.77	0.28; 1.26
Final exam	6.30 ± 1.56	6.98 ± 1.22	<0.01	0.68	0.25; 1.12
Foreign language, mean ± SD					
1st semester	7.30 ± 1.63	7.96 ± 0.93	<0.01	0.66	0.19; 1.13
Final exam	7.62 ± 1.75	8.57 ± 1.12	<0.01	0.94	0.47; 1.42
Mathematics, mean ± SD					
1st semester	5.58 ± 1.90	6.78 ± 1.52	<0.01	1.2	0.61; 1.79
Final exam	5.33 ± 1.60	6.92 ± 1.68	<0.01	1.59	1.10; 2.08
Anxiety, median (min; max)					
at baseline	3.0 [0.0; 10.0]	4.0 [0.0; 11.0]	0.05	0.7	0.08; 1.31
at follow up	4.0 [0.0; 8.0]	4.0 [0.0; 10.0]	0.6	0.24	−0.42; 0.89
Physical health, median [min; max]					
at baseline	95.0 [65.0; 100.0]	95.0 [20.0; 100.0]	0.45	−2.83	−5.36; −0.40
at follow up	95.0 [65.0; 100.0]	95.0 [20.0; 100.0]	0.97	−1.74	−4.72; 1.25
Role limitations due to physical health, median [min; max]					
at baseline	75.0 [0.0; 100.0]	100.0 [0.0; 100.0]	0.84	−0.06	−8.30; 8.18
at follow up	75.0 [0.0; 100.0]	100.0 [0.00; 100.0]	0.61	0.76	−7.59; 9.10
Role limitations due to emotional problems, median [min; max]					
at baseline	66.7 [0.0; 100.0]	66.7 [0.0; 100.0]	0.72	1.64	−9.04; 12.3
at follow up	66.7 [0.0; 100.0]	33.3 [0.0; 100.0]	0.2	−7.40	−18.6; 3.84
Energy/fatigue, median [min; max]					
at baseline	60.0 [13.3; 100.0]	60.0 [5.0; 100.0]	0.99	−0.63	−6.11; 4.86
at follow up	57.5 [15.0; 95.0]	55.0 [5.0; 95.0]	0.6	−1.90	−7.50; 3.69
Emotional well-being, median [min; max]					
at baseline	72.0 [28.0; 100.0]	64.0 [8.0; 100.0]	0.92	−3.94	−8.73; 0.85
at follow up	69.0 [24.0; 96.0]	60.0 [12.0; 100.0]	0.08	−5.08	−10.3; 0.15
Social functioning, median [min; max]					
at baseline	75.0 [0.0; 100.0]	87.50 [0.0; 100.0]	0.54	1.26	−5.00; 7.53
at follow up	75.0 [0.0; 100.0]	75.0 [12.5; 100.0]	0.24	−3.60	−10.0; 2.82
Pain, median [min; max]					
at baseline	77.5 [22.5; 100.0]	72.5 [12.5; 100.0]	0.19	−4.17	−9.45; 1.10
at follow up	77.5 [12.5; 100.0]	77.5 [20.0; 100.0]	0.46	1.83	−3.53; 7.18
General health, median [min; max]					
at baseline	67.5 [10.0; 95.0]	70.0 [5.0; 100.0]	0.92	−0.49	−5.48; 4.50
at follow up	70.0 [25.0; 100.0]	65.0 [15.0; 100.0]	0.53	1.72	−7.20; 3.77

**Table 3:** Differences in grades, anxiety, and QOL, by genders.

	Boys	Girls	*p* Value	Mean Difference	95% CI
Latvian language, mean ± SD					
1st semester	5.61 ± 1.14	6.85 ± 1.66	<0.01	1.23	0.78; 1.69
Final exam	6.29 ± 1.25	7.34 ± 1.23	<0.01	1.05	0.74; 1.37
Foreign language, mean ± SD					
1st semester	7.58 ± 1.18	7.86 ± 1.38	0.05	0.28	−0.13; 700
Final exam	8.41 ± 1.30	8.25 ± 1.43	0.4	−1.58	−0.50; 0.19
Mathematics, mean ± SD					
1st semester	5.88 ± 1.57	6.86 ± 1.86	<0.01	0.99	0.43; 1.54
Final exam	6.24 ± 1.74	6.81 ± 1.83	0.01	0.56	0.11; 1.02
Anxiety, median (min; max)					
at baseline	3.0 [0.0; 8.0]	4.0 [0.0; 11.0]	<0.01	1.27	0.67; 1.89
at follow up	3.0 [0.0; 9.0]	4.0 [0.0; 10.0]	<0.01	1.17	0.60; 1.73
Physical health, median [min; max]					
at baseline	100.0 [35.0; 100.0]	95.0 [20.0; 100.0]	<0.01	−3.47	6.23; −0.70
at follow up	100.0 [35.0; 100.0]	95.0 [20.0; 100.0]	<0.01	−3.33	−5.98; −0.69
Role limitations due to physical health, median [min; max]					
at baseline	100.0 [0.00; 100.0]	75.0 [0.0; 100.0]	0.02	−8.09	−15.6; −0.57
at follow up	100.0 [0.0; 100.0]	75.0 [0.0; 100.0]	<0.01	−7.58	−15.0; −0.17
Role limitations due to emotional problems, median [min; max]					
at baseline	100.0 [0.0; 100.0]	33.3 [0.0; 100.0]	<0.01	−28.0	−37.3; −18.9
at follow up	66.7 [0.0; 100.0]	33.3 [0.0; 100.0]	<0.01	−23.8	−33.4; −14.1
Energy/fatigue, median [min; max]					
at baseline	65.0 [15.0; 100.0]	50.0 [5.0; 100.0]	<0.01	−14.5	−19.3; −9.80
at follow up	60.0 [5.0; 95.0]	50.0 [5.0; 90.0]	<0.01	−11.5	−16.2; −6.74
Emotional well-being, median [min; max]					
at baseline	72.0 [28.0; 100.0]	60.0 [8.0; 100.0]	<0.01	−11.0	−15.2; −6.70
at follow up	68.0 [16.0; 100.0]	56.0 [12.0; 96.0]	<0.01	−11.1	−15.8; −6.34
Social functioning, median [min; max]					
at baseline	87.5 [25.0; 100.0]	75.0 [0.0; 100.0]	<0.01	−12.5	−18.1; −6.92
at follow up	87.5 [25.0; 100.0]	75.0 [0.0; 100.0]	<0.01	−11.2	−16.8; −5.62
Pain, median [min; max]					
at baseline	77.5 [22.5; 100.0]	67.5 [12.5; 100.0]	0.01	−5.48	−10.3; −0.62
at follow up	77.5 [20.0; 100.0]	67.5 [12.5; 100.0]	<0.01	−6.99	−11.7; −2.27
General health, median [min; max]					
at baseline	70.0 [30.0; 100.0]	60.0 [5.0; 100.0]	<0.01	−8.53	−13.0; −4.06
at follow up	70.0 [20.0; 100.0]	63.8 [15.0; 100.0]	<0.01	−10.2	−15.0; −5.48

**Table 4:** Highly anxious students *, by time point and by school.

Time Point	Level of Comparison	Non-Anxious, N (%)	Anxious, N (%)	*p* Value
Baseline		198 (76.4%)	61 (23.6%)	0.90
Follow up		197 (77.0%)	59 (23.0%)	
Baseline	Regular school Gymnasium	69 (86.2%) 129 (72.1%)	11 (13.8%) 50 (27.9%)	<0.01
Follow up	Regular school Gymnasium	59 (84.3%) 138 (74.2%)	11 (15.7%) 48 (25.8%)	0.06
Baseline	Boys Girls	106 (85.5%) 92 (68.1%)	18 (14.5%) 43 (31.9%)	<0.01
Follow up	Boys Girls	106 (80.9%) 90 (72.6%)	25 (19.1%) 34 (27.4%)	0.08

* Anxiety level higher than 5 for boys and higher than 6 for girls.

**Table 5:** Association between students’ marks, type of school, gender, and anxiety.

Subject Matter	Variable	Effect Estimate *β*	95% CI	*p* Value	Adjusted R^2^
**1st Semester Marks**					
Latvian language	Type of school	−0.99	−1.46; −0.52	<0.01	0.27
	Gender	−1.11	−1.60; −0.63	<0.01	
	Anxiety	0.09	−0.01; 0.18	0.06	
Foreign language	Type of school	−0.61	−1.07; −0.15	0.01	0.08
	Gender	−0.32	−0.80; 0.16	0.19	
	Anxiety	0.07	−0.02; 0.15	0.15	
Mathematics	Type of school	−1.38	−1.94; −0.82	<0.01	0.22
	Gender	−0.99	−1.58; −0.41	<0.01	
	Anxiety	0.07	−0.04; 0.18	0.21	
**Final State Exam**					
Latvian language	Type of school	−0.66	−0.99; −0.22	<0.01	0.19
	Gender	−1.05	−1.38; −0.71	<0.01	
	Anxiety	0.03	−0.04; 0.10	0.46	
Foreign language	Type of school	−0.88	−1.27; −0.50	<0.01	0.09
	Gender	0.12	−0.22; 0.46	0.49	
	Anxiety	0.02	−0.06; 0.10	0.67	
Mathematics	Type of school	−1.63	−2.14; −1.13	<0.01	0.17
	Gender	−0.62	−1.07; −0.17	<0.01	
	Anxiety	0.05	−0.02; 0.12	0.16	

## Data Availability

The data is available from the corresponding author by demand.

## Appendix A

**Table A1:** Spearman correlations between level of anxiety and student academic performance.

Time Point		Foreign Language	Mathematics
Baseline	Anxiety	0.24 **	0.25 **
	Latvian language	0.50 **	0.78 **
	Foreign language		0.50 **
	Mathematics		
Follow up	Anxiety	0.09	0.08
	Latvian language	0.28 **	0.50 **
	Foreign language		0.36 **
	Mathematics		

* Significant on 0.05 level. ** Significant on 0.01 level.

**Table A2:** Reliability of the study inventories, by time point.

	Baseline, α-Cronbach	Follow Up, α-Cronbach
Level of anxiety	0.71	0.66
Physical health	0.79	0.77
Role limitations due to physical health	0.68	0.65
Role limitations due to emotional problems	0.76	0.77
Energy/fatigue	0.82	0.80
Emotional well-being	0.83	0.85
Social functioning	0.79	0.75
Pain	0.75	0.74
General health	0.77	0.79
